# Co-administration of FVIII with IVIG reduces immune response to FVIII in hemophilia A mice

**DOI:** 10.1038/s41598-022-19392-1

**Published:** 2022-11-22

**Authors:** Sajjad Afraz, Ivan Stevic, Davide Matino, Jianping Wen, Helen Atkinson, Anthony K. C. Chan, Gonzalo Hortelano

**Affiliations:** 1 Thrombosis and Atherosclerosis Research Institute, McMaster University, Hamilton, ON Canada; 2 School of Biomedical Engineering, McMaster University, Hamilton, ON Canada; 3 Department of Pathology and Laboratory Medicine, Schulich School of Medicine and Dentistry, Western University, London, ON Canada; 4 Department of Medicine, McMaster University, Hamilton, ON Canada; 5 McMaster Immunology Research Centre, McMaster University, Hamilton, ON Canada; 6 Department of Pediatrics, McMaster University, Hamilton, ON Canada; 7 Department of Biology, School of Science and Humanities, Nazarbayev University, Qabanbay Batyr Ave 53, 010000 Nur-Sultan, Kazakhstan

**Keywords:** Haematological diseases, Immunotherapy

## Abstract

Hemophilia A is an X-linked recessive congenital bleeding disorder. Exogenous infusion of FVIII is the treatment of choice, and the development of immunoglobulins against FVIII (inhibitors) remains the major challenge in clinical management of the disease. Here, we investigated the effect of co-administration of FVIII with intravenous immunoglobulin (IVIG) on the development of inhibitors in previously untreated hemophilia A mice. A group of hemophilia A mice (C57BL/6^FVIII−/−^) received weekly injections of recombinant human FVIII (rFVIII) for twelve consecutive weeks while a second group received co-injections of rFVIII + IVIG. An in-house enzyme-linked immunosorbent assay (ELISA) was designed to detect antibodies to rFVIII. Every mouse in the first group developed antibodies to rFVIII. In contrast, mice treated with rFVIII + IVIG showed significantly lower antibody titers. Interestingly, when co-administration of IVIG was discontinued after 12 weeks in some mice (rFVIII continued), these mice experienced an increase in antibody titer. In contrast, mice that continued to receive rFVIII + IVIG retained significantly lower titers. In conclusion, prophylactic rFVIII co-administration with IVIG modulated the immune response to FVIII and resulted in decreased anti-FVIII antibody titer. These findings suggest that co-injection therapy with IVIG could potentially be effective in the management of hemophilia A patients at risk of inhibitor development.

## Introduction

Hemophilia A is defined as a deficiency of functional endogenous procoagulant factor VIII (FVIII). It is a rare genetic disorder which can affect approximately 1:10,000 live births across races and ethnic groups^1^. In the United States, around 400 babies are born with hemophilia A annually^2^. The disease is inherited in an X-linked recessive manner, but new cases can also arise from de novo mutations in approximately 30% of cases^3^. Due to its inheritance, the disease occurs mostly in males with rare exceptions in females. Generally, female individuals are asymptomatic carriers, or are mildly affected^4^.

Factor replacement therapy is the treatment of choice for hemophiliacs, which is generally well tolerated^5,6^. However, the development of inhibitors, specific alloantibodies that neutralize the infused factor, is a serious complication of the treatment which can be seen in 10–40% of patients with severe hemophilia A^4,7^. Although current factor replacement treatment is highly effective in reducing morbidity and improving quality of life in hemophilia patients, inhibitor development has remained a major challenge in the management of hemophilia patients^4,7,8^.

Several novel approaches such as gene therapy^9^, the application of novel extended half-life FVIII products^10,11^, as well as, the development of non-factor therapies such as a monoclonal antibody mimicking co-factor activity of FVIII^12^ have been assessed in recent years in order to prevent, or decrease, the risk of inhibitor formation in hemophilia patients. Although promising results have been reported from these novel treatment techniques, they suffer from limitations^13–15^.

A potential therapeutic approach to modulate the immune responses to FVIII and prevent the generation of inhibitory antibodies is the administration of FVIII products jointly with other proteins. Some studies have observed a decrease in the anti-FVIII immune response in mouse models of hemophilia A when human FVIII was administered in the presence of other proteins such as factor IX (FIX)^16^, von Willebrand Factor (vWF)^17^, or albumin^18^. These findings were attributed to the phenomenon of antigenic competition^16,19^. A decrease in the immune response to a specific antigen may occur following its administration in the presence of a modulatory antigen, as a consequence of competition by two antigens at several levels of interaction between antigen and host immune cells. This may result in fewer epitope regions from both the antigen and the modulator protein being effectively presented^20–22^. It is conceivable that a similar immunomodulatory effect may be seen when FVIII is administered together with other proteins.

Intravenous immunoglobulin (IVIG) is a concentrated antibody solution purified from pooled plasma from thousands of donors. Originally it was used as an immunoglobulin replacement therapy in immunocompromised patients to protect them against infectious diseases^23^. Following the observation of the immunomodulatory effects of IVIG^23^, it is now being used in the treatment of an increasing number of diseases such as autoimmune and inflammatory disorders, as well as, immunodeficiency disorders^23,24^. Although IVIG is widely used in clinics, its mechanism of action is not yet fully understood^25^. Several mechanisms of action have been proposed to explain the multiple beneficial effects of IVIG^23^. However, further investigation is needed in order to clarify the exact mechanism^23,25^.

Human IVIG has been shown to induce similar immunomodulatory effects in mice^26–28^, however the immunomodulation has not been studied previously in hemophilia A mice. Herein we investigated the potential effect of human IVIG co-injected with recombinant human FVIII (rFVIII) on the development of antibodies to FVIII in hemophilia A mice, hypothesizing that co-injection of FVIII together with IVIG will modulate anti-FVIII immune responses in hemophilia A mice.

## Materials and methods

### Materials and reagents

Full-length rFVIII (Kogenate® FS, Bayer, Germany) and human IVIG (Gamunex®, Grifols Therapeutics, Research Triangle Park, NC, USA) were used in this study. Goat anti-mouse IgG (H&L) alkaline phosphatase conjugated detecting antibody and mouse polyclonal anti-human FVIII antibody were purchased from Abcam Inc (Toronto, ON, Canada). p-Nitrophenyl phosphate disodium hexahydrate (p-NPP) tablets were purchased from Sigma-Aldrich (Oakville, ON, Canada).

### Animal experimentation

**Figure 1:**
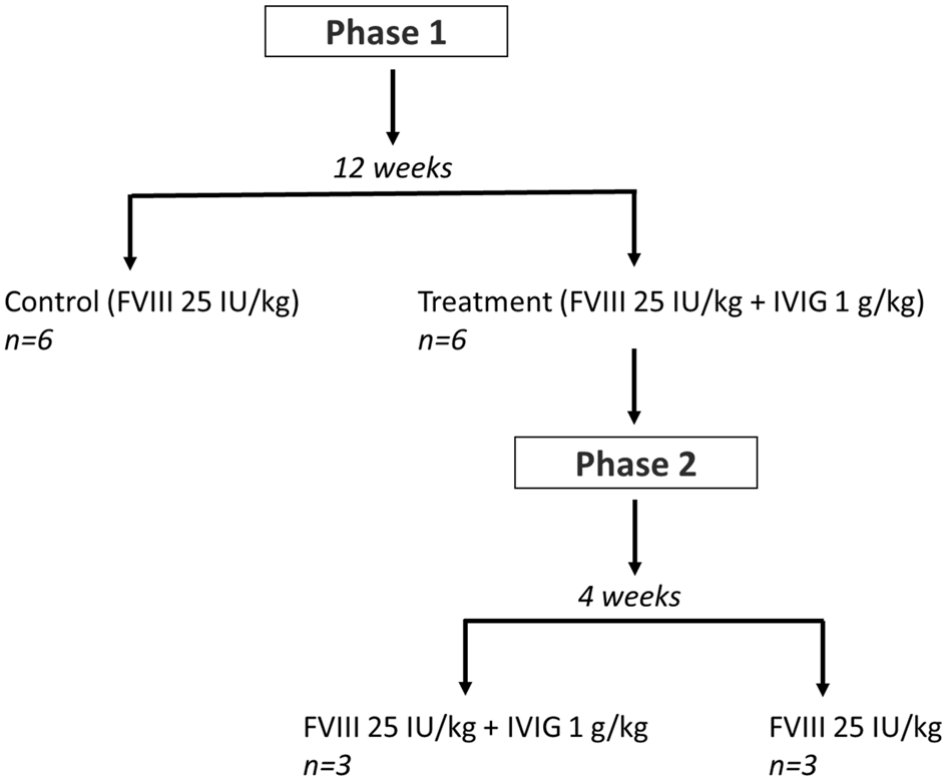
Experimental design.

C57BL6 E16 hemophilia A mice aged 8–12 weeks were used. All animal experiments were approved by the Animal Research Ethics Board at McMaster University (Animal Utilization Protocol #19-02-08), and all methods were performed in accordance with the guidelines of the Canadian Council for Animal Care. The study was also carried out in compliance with the ARRIVE guidelines. The experiment was designed in two phases (Fig. 1). In phase 1, a control group (n = 6) of previously untreated mice were immunized by weekly intraperitoneal (IP) injection of rFVIII for 12 consecutive weeks. An additional experimental treatment group (n = 6) of naive hemophilia A mice were treated with co-injection of rFVIII with IVIG for the same duration of time.

For phase 2 of the experiment, mice in the treatment group were randomly allocated to 2 additional groups (n = 3 mice per group). Mice in the first group continued to receive IP co-injections of rFVIII with IVIG for an additional 4 weeks, while mice in the second group received rFVIII alone for the same duration of time. The experimental period was 16 weeks in total. The administered dose of FVIII was 25 IU/kg, and the IVIG dosage was 1 g/kg for all injections. Blood samples of 50–100 μL were collected bi-weekly, prior to each injection, via retro orbital plexus using capillary tubes. Plasma was separated by centrifugation and stored at − 80 °C. Mouse anti-human FVIII IgG in plasma samples was detected by an enzyme linked immunosorbent assay (ELISA) as described in the following section.

Furthermore, an additional set of experiments was conducted to determine if the same dosage of IVIG could also modulate the immune response against a higher dosage of rFVIII (100 IU/kg instead of 25 IU/kg). Naïve hemophilic mice were injected weekly with either rFVIII plus IVIG (n = 12) or rFVIII alone (n = 12) for 4 weeks. Plasma samples were collected and anti-FVIII antibody levels analysed by ELISA. After 4 weeks, mice in the rFVIII plus IVIG treatment group were randomly divided into two additional groups, where they were either injected with the high dose rFVIII alone (Treatment 1) or co-injected with high dose rFVIII plus IVIG 1 g/kg (Treatment 2) for an additional 8 weeks. Mice in the original rFVIII alone group continued to be injected weekly with rFVIII for an additional 8 weeks (control, n = 6). Plasma samples were collected bi-weekly and anti-FVIII antibody levels analysed by ELISA.

### Anti-FVIII antibody ELISA

A modified ELISA was developed to detect antibodies to rFVIII in mice based on a previously published protocol^29^. Wells of ELISA strips (Corning® clear polystyrene high bind Stripwell, Corning, NY, USA) were coated with 50 ng of rFVIII in 100 μL coating buffer (0.1 M Na_2_CO_3_, 0.1 M NaHCO_3_, pH 9.6) and the plate was covered and incubated for 2 h at room temperature. The wells were washed 3 times with phosphate buffered saline containing 1% Tween 20 (PBS/T). Tween 20 was added for more effective washing, to prevent non-specific background staining. Blocking buffer (5% skim milk solution in PBS/T) was applied and the wells were covered and incubated for another 2 h at room temperature, and then washed as described above. Plasma samples were diluted 1:100 in blocking buffer and 100 μL of each diluted sample was applied to each well. The wells were covered and incubated over night at room temperature. The wells were then washed as above, and the detecting antibody (goat anti-mouse IgG (H&L) conjugated to alkaline phosphatase) was diluted 1:1000 in blocking buffer and 100 μL of the diluted antibody solution was applied. Diluting the antibody in blocking buffer helped to minimize non-specific binding and background. The wells were covered and incubated 2 h at room temperature and then washed as before. The developing solution was prepared by dissolving one p-NPP 5 mg tablet per 5 mL DEA buffer (1 M Diethanolamine, 0.5 mM MgCl_2_, pH 9.8), and 100 μL of developing solution was placed into each well. The color reaction was stopped after 20 min by adding 50 μL of 3 M NaOH per well. The absorbance in each well was measured at a wavelength of 405 nm using a SpectraMax® Plus 384 microplate reader (Molecular Devices, San Jose, CA, USA). This antibody ELISA had an optical density (OD) detection limit of 0.2, values below the limit of detection were treated as zero. The limit of detection was calculated using a formula based on a previously published study^30^.

A standard curve was generated to quantify the results. Commercial mouse polyclonal anti-human FVIII antibody was used as a standard on each ELISA plate. Two series of two-fold serial dilutions (2.5 μg/100 μL–0.15 μg/100 μL) of the commercial antibody were included with each performance of the ELISA. The average absorbance value of each set of duplicate standards was calculated, and a standard curve was created by plotting the mean absorbance (y axis) against the corresponding dilution (x axis) and drawing a best fit trend line using a 4-parameter logistic (4PL) regression model in SigmaPlot (Systat Software, San Jose, CA, USA).

### Anti-IVIG antibody ELISA

A similar ELISA method was used to detect mouse anti-human IVIG antibody. Briefly, wells were coated with 100 ng/well of human IVIG in 100 μL of coating buffer. Blocking steps were undertaken following the incubation period, and the washing steps, sample applications and incubations were as described above. 100 μL of a 1:10,000 dilution of the detecting antibody was applied to each well and incubated as described above. The wells were washed and 100 μL of developing solution was added to the wells. The reaction was stopped after 20 min by adding 50 μL of 3 M NaOH and the absorbance was measured at 405 nm. The OD detection limit was 0.46.

### Bethesda assay

Plasma samples were analyzed for FVIII inhibitory antibodies using the Nijmegen modification of the Bethesda assay^31^. Briefly, blood samples of 600–800 μL were collected through cardiac puncture and plasma was separated by centrifugation. Heat deactivating step was then undertaken by incubating plasma samples in 56 °C to degrade any remaining FVIII protein in the samples that may interfere with the assay. After 30 min, samples were recentrifuged and supernatant was collected. In the next step, two-fold serial dilutions (1/2–1/16) of collected samples were prepared using imidazole buffer (0.05 M imidazole, pH 7.4), and test mixture was made by preparing 1:1 mixture of buffered pooled normal plasma (buffered-PNP; 0.1 M imidazole, pH = 7.4, FVIII 95–110%) with either undiluted or pre-diluted samples. Control mixture was made by preparing 1:1 mixture of buffered-PNP with imidazole buffer. Both test mixture and control mixture were incubated for 2 h at 37 °C in water bath followed by a 10 min incubation on ice. FVIII coagulant (FVIII:C) activity of both mixtures was then measured using one stage APTT-based clotting assay, and the residual FVIII:C activity was calculated as relative percentage FVIII: C activity of the test mixture compared to the control mixture (RA%). Bethesda unit was calculated using the equation [2-LOG(RA%)]/0.31003. When the residual FVIII:C activity of test sample was below 25%, test was reperformed using more diluted sample until a residual FVIII:C activity of 25–75% was achieved.

### Statistical analysis

Data are expressed as mean and SEM. Graphs were produced in Prism (GraphPad, San Diego, CA, USA). Comparisons of the data were performed using IBM SPSS Statistics 22 using Student’s t-test unless otherwise specified, and *P* < 0.05 was considered to be statistically significant.

## Results

### Prophylactic effect of FVIII and IVIG co-injection on inhibitor development

**Figure 2:**
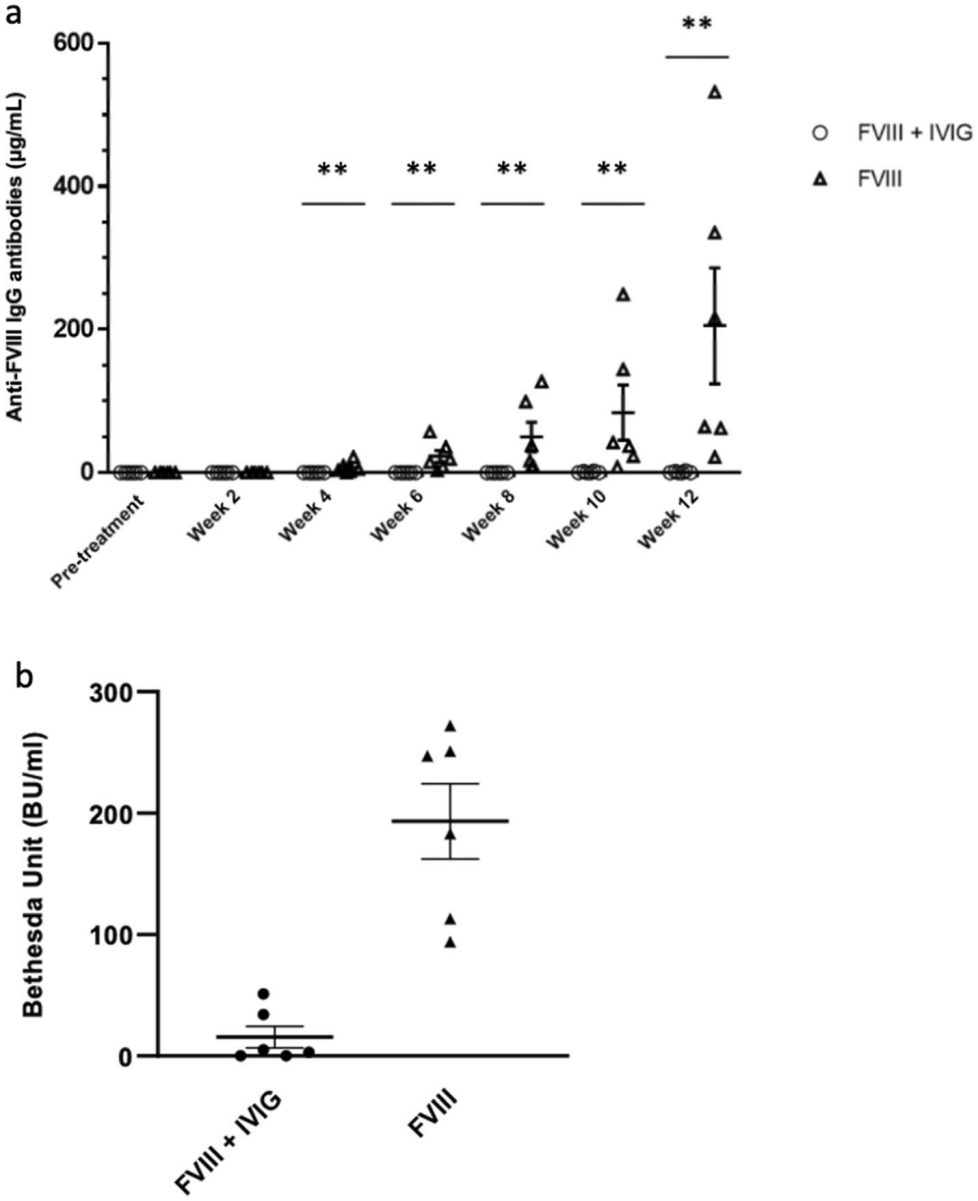
(**a**) Comparison of anti-FVIII antibody levels between treatment and control groups after repeated weekly IP injections for 12 weeks. Hemophilia A mice in the treatment group received FVIII co-injected with IVIG (n = 6). Hemophilia A mice in the control group received FVIII alone (n = 6). Plasma samples were collected pre-treatment and then bi-weekly 1 week after the last injection. Anti-FVIII antibody levels were measured via ELISA. Horizontal bars and error bars represent mean ± SEM. **P < 0.01 for the treatment group vs. the control group. (**b**) FVIII inhibitory activity in hemophilia A mice measured by the Nijmegen modification of the Bethesda assay following treatment with FVIII (n = 6) or FVIII + IVIG (n = 6). The difference between the means is statistically significant (*P* < 0.01).

The effect of co-injection of rFVIII with IVIG on the development of anti-FVIII antibodies in previously untreated hemophilia A mice was evaluated, and compared to a control group of mice that received rFVIII alone (Fig. 2). Anti-FVIII antibodies were detectable after 4 weeks in most (83%) mice injected with rFVIII alone (control), and by week 6 in all the control mice (Fig. 2a), with a titer that continued to increase until the end of the experiment in week 12. The mean anti-FVIII antibody levels after 4 and 12 weeks was 6.98 µg/mL and 205.2 µg/mL, respectively. Compared to mice that received rFVIII alone, mice that were co-injected with rFVIII and IVIG had lower antibody levels during the entire 12 weeks of treatment with a mean anti-FVIII antibody titer of 0.65 µg/mL after 12 weeks (Fig. 2b). This titer is significantly different from the 205.2 µg/mL in the control group (*P* < 0.01)*.* In addition to a lower level of total antibodies, our results also show that the mice in the treatment group had significantly lower anti-FVIII inhibitor levels compared to the control mice, as measured by the modified Bethesda assay (Fig. 2b). Notably, while all animals in the control group produced anti-FVIII antibodies, only 2 out of 6 mice in the treatment group developed detectable antibodies against FVIII (starting at week 10). Furthermore, the antibody levels in these 2 individual mice were lower than those in the control group. Therefore, co-injection of rFVIII with IVIG significantly reduced and delayed the anti-FVIII immune response in previously untreated hemophilia A mice.

### Effect of IVIG cessation on anti-FVIII antibody level

**Figure 3:**
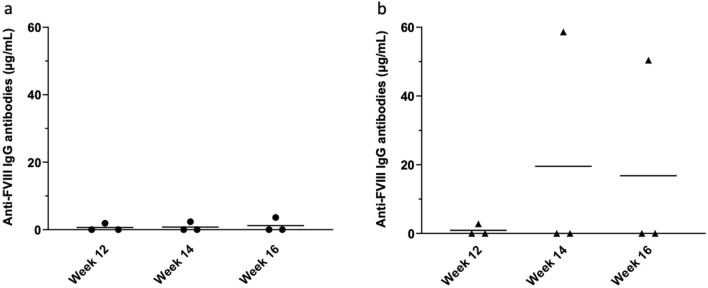
Anti-FVIII antibody development pattern of each mouse in the treatment group after receiving weekly injection of (**a**) FVIII + IVIG or (**b**) FVIII alone for the additional duration of 4 weeks following 12 weeks of co-injection therapy. Plasma samples were collected and anti-FVIII antibody levels analysed as described in Fig. 2. Horizontal bars represent mean.

An important question is whether the modulatory effect observed due to the co-administration of IVIG is maintained if the IVIG administration is discontinued. In order to further investigate the effect of the treatment, mice in the treatment group were randomly divided into two groups in phase 2 of the experiment, after having received co-injection of rFVIII with IVIG for the duration of 12 weeks during the first phase. While 3 mice continued to receive rFVIII and IVIG co-injection weekly for another 4 weeks, the remaining 3 mice received only rFVIII for the same 4 weeks. IVIG was removed from the treatment regimen in the latter group in order to investigate the effect of stopping IVIG injection on anti-FVIII antibody level. We hypothesized that 12 weeks of prophylactic co-injection therapy with IVIG would induce immune tolerance to FVIII in hemophilic mice, such that they would not produce anti-FVIII antibodies when exposed to FVIII in the absence of IVIG. Figure 3 indicates the anti-FVIII antibody level of each mouse in phase 2. While the antibody level remained undetectable in 2 mice after IVIG was removed from the treatment regimen, one mouse developed high amounts of anti-FVIII antibody. Interestingly, the response to IVIG withdrawal correlated with the preexistence of antibodies during the preceding 12 weeks of co-injection therapy; mice that had undetectable levels of anti-FVIII antibodies remained unresponsive after IVIG was removed from the treatment, while the mouse with low but detectable antibodies developed high amounts of anti-FVIII antibody once the IVIG injections ceased. Similar results were seen in the other group; 2 of 3 mice did not produce antibodies following further co-injection of rFVIII and IVIG for 4 weeks, and one mouse that already had low antibody levels continued to produce antibodies, albeit the level remained relatively low. Accordingly, the effect of IVIG persisted following the cessation of co-injection therapy and the results suggest a possible modulatory effect of IVIG on the development of antibodies to FVIII. However, the small sample size does limit our ability to draw a definitive conclusion from these results.

### Evaluation of potential interference of IVIG with anti-FVIII antibody ELISA

In order to evaluate possible binding and interactions between human polyclonal IgG contained in mouse plasma and coated rFVIII protein in ELISA assay, and to rule out the possibility that those potential interactions would affect the obtained results, a separate ELISA assay was designed. The well was first coated with rFVIII. Next, human IVIG was applied to the well. The detecting antibody was then applied, and absorbance was measured at a wavelength of 405 nm. The obtained OD was at background level, equivalent to the negative control. Therefore, human IVIG did not crosslink to coated rFVIII in the anti-FVIII antibody ELISA.

An additional assay was performed to specify how the presence of IVIG in the test sample may affect the measured OD. Wells were coated with rFVIII and a plasma sample expected to contain a high amount of anti-FVIII antibodies (positive control) was applied to a well. In another rFVIII-coated well, the same plasma sample plus human IVIG were applied. The detecting antibody was added, and absorbance was measured at a wavelength of 405 nm. No difference was seen in the OD values obtained from the two test samples. Therefore, the presence of IVIG molecules in the test environment did not cause a significant difference in the OD values obtained in the anti-FVIII antibody ELISA.

### Anti-IVIG antibody development in mice

**Figure 4:**
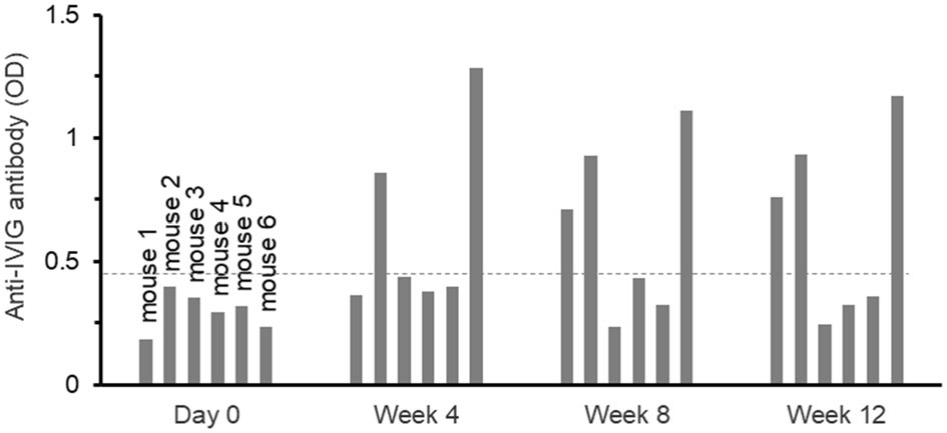
Anti-human IVIG antibody levels of each mouse following co-injection therapy in the treatment group determined by ELISA. Data are presented as the measured OD_405_ from the ELISA assay. The dashed line represents the limit of detection, any sample with an OD below that point is considered negative for the presence of anti-IVIG antibodies.

It is plausible that the modulation exerted by IVIG on the development of antibodies to FVIII also leads to the development of antibodies to IVIG in the treated mice. In order to assess the immune response against injected human IVIG in the treatment group, the amount of anti-IVIG antibody in each mouse during the 12 weeks of co-injection treatment was measured by ELISA (Fig. 4). Overall, 3 out of 6 mice, including 2 mice that had shown some degree of anti-FVIII antibody development, had detectable anti-IVIG antibodies. Interestingly, mouse #6 that had a high level of anti-IVIG antibodies also developed the highest anti-FVIII antibody level in the treatment group (Fig. 4).

### Effect of co-injection therapy with a higher dose of FVIII

**Figure 5:**
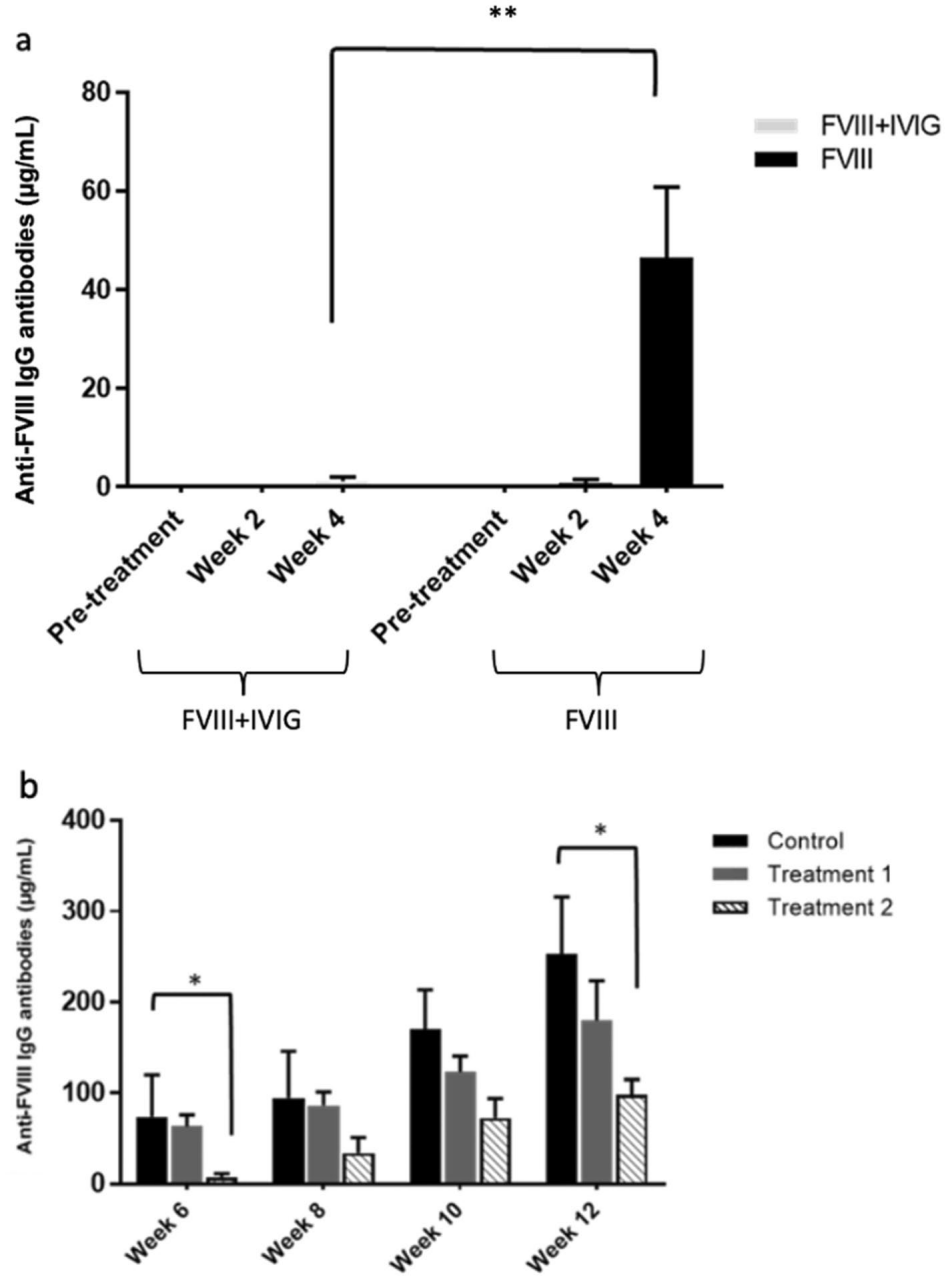
Effects of high dose FVIII (100 IU/kg) co-injected with IVIG on anti-FVIII antibody development in previously untreated hemophilia A mice. (**a**) Hemophilic mice were injected weekly with either FVIII + IVIG (n = 12) or FVIII alone (n = 12) for 4 weeks. Plasma samples were collected and anti-FVIII antibody levels analysed as described in Fig. 2. Data are presented as mean and error bars represent the SEM. **P < 0.01 for treatment vs. control. (**b**) The mice in the FVIII + IVIG treatment group were randomly divided into two groups termed Treatment 1 (n = 6) and Treatment 2 (n = 6), and they were treated weekly either with 100 IU/kg FVIII alone (Treatment 1) or 100 IU/kg FVIII co-injected with IVIG (Treatment 2) for an additional 8 weeks. Mice in the original FVIII alone group continued to be injected weekly with FVIII for an additional 8 weeks (control, n = 6). Plasma samples were collected and anti-FVIII antibody levels analysed as described in Fig. 2. Data are presented as mean and error bars represent the SEM. One-way ANOVA followed by Tukey’s post hoc test was performed to determine the significance of difference between the groups. Significant differences are indicated as *P < 0.05.

Weekly injections of a higher dose of rFVIII (100 IU/kg) resulted in higher and earlier development of anti-FVIII antibodies in the control group of mice treated with rFVIII alone (Fig. 5a) and all of these mice produced detectable antibody levels by week 4. Compared to the control mice, only 3 out of 12 mice in the treatment group had detectable anti-FVIII antibody at week 4 and the average antibody level was lower and significantly different in the treatment group compared with the control (Fig. 5a; mean anti-FVIII antibodies of 1.26 µg/mL compared to 46.58 µg/mL after 4 weeks in the treatment and control groups respectively. *P* < 0.01).

After 4 weeks, mice in the treatment group were either injected with the high dose rFVIII alone (Treatment 1) or co-injected with high dose rFVIII plus IVIG 1 g/kg (Treatment 2) for an additional 8 weeks. Figure 5b illustrates the results. All mice in the Treatment 1 group developed antibodies against FVIII following the removal of IVIG from the treatment, while the antibody level remained undetectable in 2 out of 6 mice from the Treatment 2 group by week 6. There was no statistically significant difference in mean antibody levels of the Treatment 1 group compared to the control group treated with only rFVIII from the beginning. Although all mice in the Treatment 2 group developed anti-FVIII antibody by week 8, the levels of antibody were lower compared to the control during the whole experimental period and this difference was statistically significant at week 6 and week 12 (*P* < 0.05). The results indicate that the presence of IVIG had modulatory effects reducing the antibody immune responses against FVIII even after increasing the dose of injected FVIII by fourfold to 100 IU/kg.

**Figure 6:**
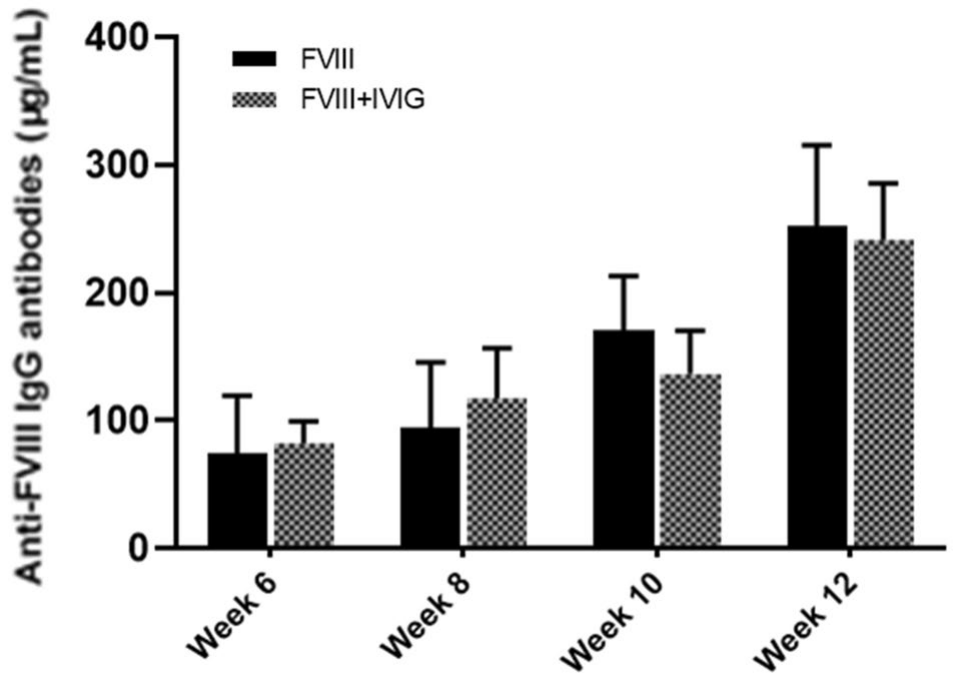
Effect of FVIII and IVIG co-injection on anti-FVIII antibody development in previously immunized hemophilia A mice. First, mice in both groups were immunized with weekly FVIII injections for 4 weeks. All mice had detectable anti-FVIII antibodies at this point. Then, mice in the original FVIII alone group continued to be injected weekly with FVIII for an additional 8 weeks while mice in the other group were treated with co-injection of FVIII with IVIG weekly. Plasma samples were collected and anti-FVIII antibody levels analysed as described in Fig. 2. Data are presented as mean and error bars represent the SEM. The differences between two groups are not significant (n = 6).

To assess the effect of the treatment on pre-existing anti-FVIII antibodies, a group of mice were treated with rFVIII and IVIG co-injection for the duration of 8 weeks after being immunized with weekly injections of high dose rFVIII for 4 weeks (Fig. 6). rFVIII and IVIG co-injection did not reduce the levels of previously developed anti-FVIII antibodies in the immunized mice. All treated mice continued to develop anti-FVIII antibodies despite being treated with co-injection of rFVIII with IVIG (Fig. 6).

## Discussion

The aim of this project was to assess whether co-administration of FVIII with IVIG would alter the level of humoral (antibodies) immune response to FVIII in hemophilia A mice. The hypothesis was that infusions of FVIII protein together with IVIG would inhibit the development of antibodies to human FVIII in naive hemophilia A mice. The overall findings from this study support our initial hypothesis, that, IVIG co-injected with FVIII reduces anti-FVIII antibody development in previously untreated hemophilia A mice, with the caveat that we were working with a small sample size. A longer follow-up might also have allowed a clearer conclusion.

It has previously been shown that hemophilia A mice develop antibodies in response to repetitive rFVIII injections^32^. In our experimental model, weekly injection of 25 IU/kg rFVIII led to the development of anti-FVIII antibodies in all previously untreated hemophilia A mice that continued to increase in titer until the end of the experiment (week 12), in agreement with published data^16,32^ (Fig. 2). The antibody was detected earlier and at a higher titer when rFVIII was injected weekly at a higher dose of 100 IU/kg (Fig. 5a). This model is reproducible, and thus suitable for studying inhibitor development in hemophilia A mice. It is known that the amount of antigen is one determining factor in stimulating host immune response and antibody production^33^. In our study, inhibition of anti-FVIII immune response was achieved more effectively when the rFVIII injection dose was 25 IU/kg, which is within the dose range being used in the management of hemophilia A patients in clinical settings^6^.

Modulation of immune response to human FVIII by co-administration of other antigens has been previously reported^16,17^. Qadura et al., compared immune responses to human plasma- derived and recombinant FVIII in hemophilia mice^16^. They treated hemophilia A mice with 4 weekly intravenous injections of 80 IU/kg FVIII either alone or mixed with human FIX. Mice generated significantly lower anti-FVIII antibody when infused with FVIII and FIX. Interestingly, they did not observe high anti-human FIX antibody. The authors suggested that antigenic competition might be responsible for the observed effect. A reduction in FVIII inhibitor levels was also reported in a study comparing immunogenicity of different human FVIII products in hemophilia A mice^17^. Mice that received FVIII products containing high amounts of human-vWF developed lower anti-FVIII antibody levels compared with mice treated with FVIII products without vWF. The murine immune system recognizes secondary proteins (vWF or recombinant human FIX) contained in FVIII products and therefore competition occurs in recognition and presentation of two antigens to the immune cells, and consequently influences the development of the immune response against FVIII^19^. Previous studies on antigen competition emphasize that antigen competition occurs mainly at the level of peptide binding to major histocompatibility complex (MHC) molecules on antigen presenting cells (APCs)^20,34^. As it has been described, in the presence of an appropriate amount of a secondary antigen, related and unrelated peptides compete to bind to the MHC binding sites. As a result, fewer peptides from each antigen are presented on the surface of the APCs, resulting in fewer T cells primed, and thus decreased antibody production^20^. In our study, human IVIG may act as a secondary antigen along with human FVIII. Thus, it is conceivable that, the concomitant presence of IVIG protein attenuated anti-FVIII antibodies through antigenic competition. It is worth pointing out that the IVIG dosage used was 1 g/kg, which mimics the dose used for the treatment of inflammatory and autoimmune diseases in humans.

Furthermore, this hypothesis may also partially explain the inability of IVIG to inhibit anti-FVIII antibodies in mice with pre-existing antibodies to FVIII (Fig. 6). Presumably, the proliferation of the cognate T cells had already occurred, and specific high-affinity antibody producing B cells as well as memory cells had been formed following the prior immunization^33,35^. Once established, these immune pathways would overcome the protective effects from antigenic competition, and as a result it would be challenging to reduce or eliminate the existing inhibitors. This would explain why our co-injection of FVIII with IVIG was not effective in the inhibition of pre-existing anti-FVIII antibodies.

The immunomodulatory effects of IVIG could also play a role in the protective effect seen in our study. There are several proposed potential mechanisms of action to explain the immunoregulatory effects of IVIG^23,25,36^. One proposed mechanism is its effect on the expansion of regulatory T cells (Tregs)^23^. In several mouse models , IVIG induced proliferation of Tregs in the spleen^26,37^. Tregs are effective immune cells in immunoregulation^38,39^. Tolerance induction by stimulating the proliferation of antigen-specific Tregs is a potential therapeutic target in the regulation of anti-FVIII reactive immune response. Adding to this, there is an epitope for Tregs called tregitope located on the IgG molecule^40^. It has been shown that tregitopes have therapeutic effects on mouse models of autoimmune diseases through expansion of CD4 + CD25 + FOXP3 + Tregs^37,41^. In the same manner, induction of Treg proliferation could potentiate the modulation of anti-FVIII immune response in hemophilia A mice as was seen in our study: two of three FVIII + IVIG treated mice did not develop anti-FVIII antibody even after discontinuation of IVIG (Fig. 3). The half-life of IgG in mice is approximately 8 days^42^, well below the immune regulatory effect observed by the IVIG treatment. Therefore, the development of active tolerance to FVIII in treated mice through expansion of FVIII-specific Tregs ought to be considered.

Our data also reflects the protective effect of IVIG when mice were exposed to a high dose of FVIII (Fig. 5). Discontinuation of IVIG from the treatment regimen after 4 weeks resulted in an increase in anti-FVIII antibody generation in mice such that there was no significant difference compared with that of the control (Fig. 5b). In contrast, The FVIII antibody level in mice that continued to receive rFVIII and IVIG co-injections was lower compared to the control during the 12 weeks of the experiment (Fig. 5b). Although the antibody level gradually increased in this treatment group, the inhibitory treatment effects lasted longer compared to the mice in which IVIG was discontinued. Thus, the reduction of FVIII antibodies observed can be ascribed to the presence of IVIG. A longer study including a larger number of mice would be necessary to better characterize the length of time necessary for IVIG to establish its immunomodulatory effect, as well as the maximum dose of rFVIII that can be tolerated.

Taken together, our results suggest that addition of IVIG to FVIII has potential immunomodulatory effect and inhibits the immune response targeted against FVIII in hemophilic mice. IVIG is an approved treatment for a variety of diseases that involve immune deficiency and is also being widely used off-label in several additional clinical conditions^43^. It is generally well tolerated, and its adverse effects are well known^43,44^. This can increase the likelihood of translating these findings to a clinical setting. Still, further studies should be conducted to determine the mechanisms behind the effects seen in our study and to assess any potential tolerance induced by the treatment. Isotyping immunoglobulins, determination of the FVIII-specific Tregs and characterization of cytokines will help unveil the type of immune response induced by the treatment, as well as assess the potential development of regulatory cytokines.

The IVIG solution used was of human source and clinical grade. The immunomodulatory effects of human IVIG in mice have been shown in several studies^26–28^. Nevertheless, one is tempted to speculate that mouse-derived immunoglobulin is likely to have an even more significant immunoregulatory effect in mice^26,36^. In our experiment, antibodies to human-immunoglobulin were detected in 3 mice from the experimental group that received IVIG (Fig. 4). Two of the three mice also developed anti-FVIII antibodies (Fig. 4, mouse 1 and 6). Interestingly, we found that the mice that developed a significantly high immune response against human IVIG also had a high anti-FVIII immune response. Development of high levels of anti-IVIG antibodies from an early stage of the experiment (as was seen in mouse 6 in Fig. 4), may have neutralized the protective effect of the infused IVIG and resulted in the development of antibodies to FVIII in those mice. The immunomodulatory effect of human IVIG observed in mice would suggest that the treatment of mice by co-administration of FVIII with mouse immunoglobulin rather than a human molecule is a worthwhile endeavour.

One limitation in this study was the variation in anti-FVIII immune responses seen among mice from the same group. The experimental mice were highly inbred, therefore mice from the same group which had received the same treatment were expected to share identical immune system characteristics and exhibit very similar immune responses. In this project, however, there were 1–2 mice in each group that produced notably different results, and the detected antibody levels were not consistent (higher or lower) compared to other mice in the group. This may be because of the complex immunology of inhibitor generation. Inhibitors are generated as a result of a cascade of interactions between different cells of the innate and the adaptive immune systems^35,45^, and any mutation or event that modulates the activation or migration pattern of immune cells will therefore potentially influence the risk to develop inhibitors^46^. Additionally, variations resulting from microenvironmental heterogeneity among mice could be another explanation for these differences. Further experimentation with a larger cohort of mice would be required for better characterizing the observed immune mechanism.

In conclusion, FVIII co-administered with IVIG can inhibit the development of antibodies to FVIII in previously untreated hemophilia A mice, suggesting that IVIG co-administration altered the immune response and alleviated FVIII inhibitor generation. Thus, co-administration of FVIII with IVIG could potentially be an effective prophylactic strategy in the management of hemophilia patients at high risk of inhibitor development. However, the exact mechanism of the immunoregulatory effect of the treatment remains unclear. Considering the fact that various immune cells and pathways are involved in the development of FVIII inhibitors, further studies should be conducted to determine the mechanisms contributing to this effect.

## Data Availability

All data generated or analysed during this study are included in this published article.
